# Solitary langerhans cell histiocytosis in an adult: case report and literature review

**DOI:** 10.1186/s13104-015-1799-z

**Published:** 2016-01-09

**Authors:** Cíntia Ferreira Gonçalves, Marília Oliveira Morais, Rita de Cássia Gonçalves Alencar, Aline Carvalho Batista, Elismauro Francisco Mendonça

**Affiliations:** Avenida Teotônio Segurado, Cj. 01, Lt. 01, Sl 508, Plano Diretor Sul, Palmas, Tocantins CEP 77061-002 Brazil; Faculdade de Odontologia, Praça Universitária S/N, Setor Universitário, Universidade Federal de Goiás, Goiânia, Goiás CEP 74605-220 Brazil; Hospital Araújo Jorge, Goiânia, Goiás Brazil

**Keywords:** Langerhans cell histiocytosis, Adult, Differential diagnosis, Treatment

## Abstract

**Background:**

Langerhans cell histiocytosis (LCH) is a disease that often affects children, but can also occur in adults and smokers. Oral manifestations are unusual and are characterized by bone pain, tooth mobility, necrotic ulcers and local edema. The aim of this paper is to describe a clinical case of LCH in an oral cavity that mimicked oral squamous cell carcinoma.

**Case presentation:**

A male, 63 years old, complaining about a “wound in the mouth” for 6 months, without any pain or spontaneous bleeding. His medical history was free of disease. The patient was a smoker for 33 years. Intraoral examination revealed a destructive ulcerative lesion around the upper left first and second molars that resembled an oral squamous cell carcinoma. Biopsy of the ulcerative lesion was performed and the microscopic features showed an inflammatory infiltrate rich in plasma cells. Based on this microscopical finding, the final diagnosis was periodontal disease associated with a proliferative non-neoplastic lesion. The patient was referred to a specialized dental surgeon and underwent periodontal therapy including surgical procedures. After that, according to follow-up with the patient, there were no signs of disease remission. The lesion increased in size, although the patient did not complain of any symptoms. A second biopsy was performed and the microscopic features again showed a rich inflammatory infiltrate with mononuclear cells and histiocytic cells, characterized by pale histiocytes with lobed nuclei, resembling a bean. A varying number of eosinophils also were observed, without any evidence of atypical cells present in this infiltrate. An immunohistochemical staining panel was done to determine the nature of this inflammatory infiltrate by using antibodies S-100, CD1a, CD-68 and CD45RO that were positive. These immunohistochemical findings were fundamental for the final diagnosis of LCH. The treatment included surgical extraction of all superior teeth, radiation and systemic corticoid therapies. After 8 years of treatment, the patient is free of disease.

**Conclusion:**

Although LCH is an unusual lesion in an oral cavity, it can be present. Biopsy and a histological exam are essential to establish the diagnosis. Immunohistochemicals were fundamental to exclude malignant lesion and to confirm the diagnosis of LCH.

## Background

Langerhans cell histiocytosis (LCH) is a disease caused by the accumulation and proliferation of abnormal bone marrow-derived Langerhans cells. These dendritic cells, lymphocytes, eosinophils and non-dendritic histiocytes form the typical infiltrates of LCH. These cells may be found at different degrees in several organs [1]. The Histiocyte Society proposed the reclassification of histiocytoses after the advent of ultrastructural studies and immunohistochemical staining [2, 3]. Moreover, proinflammatory cytokines and chemokines play a role in LCH, which suggests that it is an immune disorder. However, the detection of the oncogenic BRAF mutation in more than half of LCH patients suggests that it is a neoplastic disorder [4]. The occurrence of LCH in an oral cavity is rare; however, there are reports of LCH in oral mucosa and the most common site in the oral cavity of adults is the jaw [3, 4]. The purpose of this paper is to describe a rare case of LCH located in upper maxilla and upper oral mucosa. The clinical findings and the histological criteria were enabled to establish the final diagnosis of LCH, excluding periodontitis and carcinomatous lesion, and are presented and discussed.

## Case presentation

**Fig. 1:**
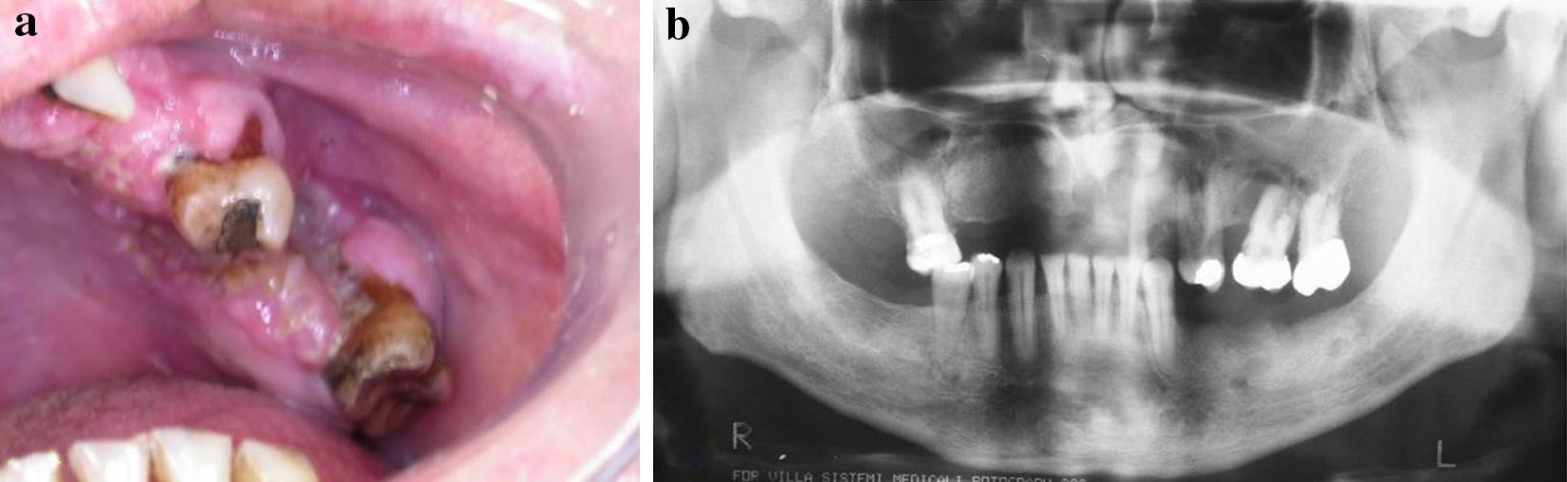
Intraoral (**a**) and panoramic radiograph (**b**) showed a lesion around the upper left first and second molars

A 63-year-old male complaining about a “wound in the mouth” was referred to Goiás Oral Medicine Center, Federal University of Goiás Dental School, Goiânia, Brasil, for diagnosis and treatment. The patient reported that the lesion had approximately 6 months of evolution and that he was asymptomatic and had not experienced any bleeding. His medical and dental history did not show any significance or any relation to his lesion. The patient had been a smoker for 33 years. Intraoral examination revealed a destructive ulcerative lesion around the upper left first and second molars, which showed mobility and poor dental conditions, including dental caries, gingival recession and bone loss (Fig. 1a). A panoramic radiograph revealed a radiolucent area in the periapical region of the upper left first molar, suggesting an osteolytic lesion (Fig. 1b). According to the clinical and radiographic features, the main diagnostic hypotheses were periodontal disease associated with oral squamous cell carcinoma. The patient reported that there was no cancer in his family history. The results of laboratory analyses, including blood and urine studies, were within the normal range. We also performed a biopsy to provide histopathological analysis. At this time, the patient took prescribed antibiotics for 7 days and underwent surgery of the upper left primary molar. Microscopic findings showed an inflammatory infiltrate rich in plasma cells that is commonly present in periodontal disease, without the presence of any atypical cell in this specimen of oral mucosa. Based on these findings, periodontal disease was considered as the diagnosis and the patient underwent periodontal therapy twice without remission of the initial injury. During follow-up, it was observed that the lesion increased considerably.

**Fig. 2:**
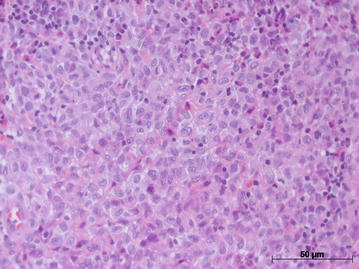
Hematoxylin-eosin staining showed basophilic nuclei and eosinophilic cell plasma

**Fig. 3:**
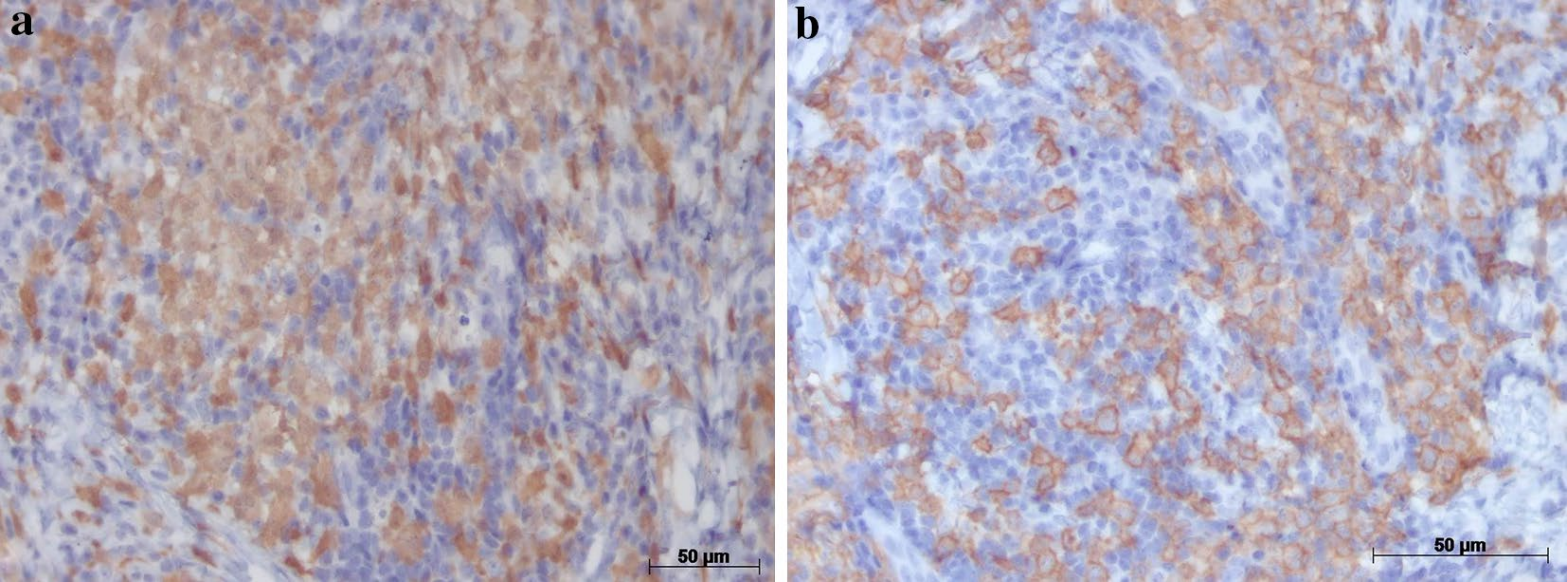
Immunohistochemical staining showed cells positive for S-100 protein (**a**) and CD1a protein (**b**)

**Fig. 4:**
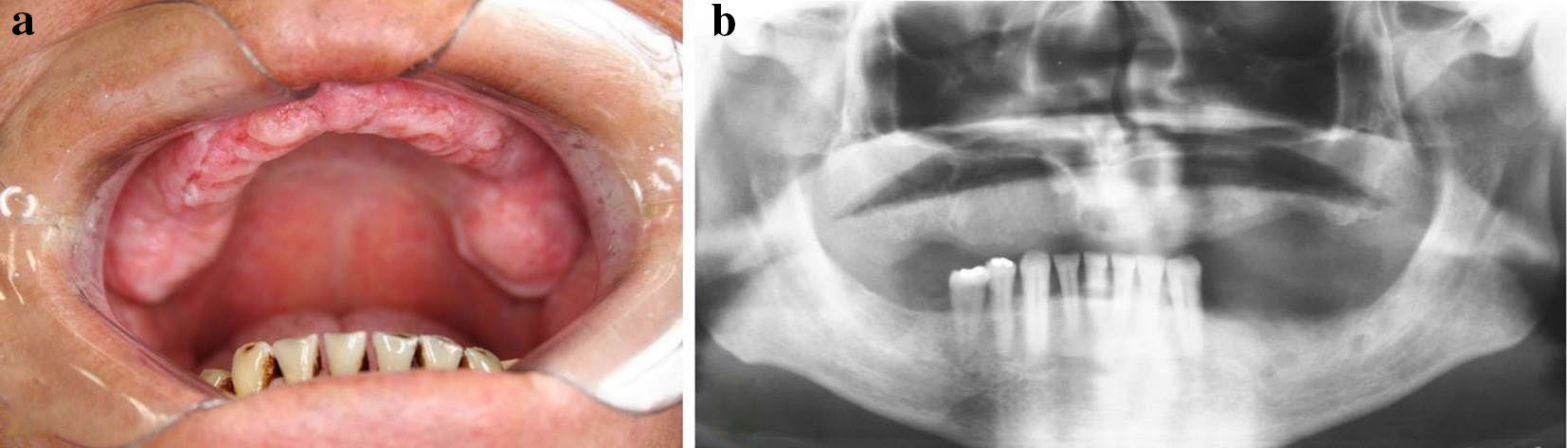
Intraoral (**a**) view and panoramic radiograph (**b**) showed no bone disease (8 years later during follow-up)

Since the lesion was increasing in size, a second biopsy was performed on a deeper level of histological plan. It showed a proliferation of cells resembling histiocytes with an occasional pale nucleus edentulous with a lobular, grain bean-like appearance (Fig. 2). Microscopic findings and the presence of histiocytic cells led to the performance of an immunohistochemical staining panel using monoclonal antibodies for S-100 (Fig. 3a), CD1a (Fig. 3b), pan-cytokeratin, CD-68, melanoma marker (HMB45) and melanoma A protein. There was positivity for S-100, CD1a, CD-68 and CD45RO (Table 1). Immunohistochemical analysis led to the diagnosis of LCH. We performed a complete investigation, including bone scintigraphy bone, abdomen CT scan; hematological exams, including blood glucose (due to diabetes risk) and a pathological analysis of bone marrow, which was hypercellular without evidence of atypical cells. The liver, spleen and lymph nodes were free of disease. There was no bone involvement besides upper maxilla. The treatment included surgical extraction of all superior teeth, due their periodontal involvement, radiation therapy, corticoid therapies and prosthesis. An established radiation protocol of 26 Gy fractionated over 13 sessions was instituted and well tolerated. We have been following up with the patient for 8 years (2006–2014) without signs of disease recurrence (Fig. 4a and b). At the last follow-up, the patient was considered “disease-free”. Hematological exams, as well as abdomen and chest exams, showed normal conditions.

**Table 1:** Immunohistochemical panel of monoclonal antibodies used in this study

Antigen	Antibody	Dilution	Specificity	Results
S-100	Lyophilized polyclonal, biocare	1:400	Langerhans cells, cells of the peripheral nervous system, melanocytes and tumor cells derived from these cell lines	+
CD1a	Monoclonal, novocastra	1:600	Langerhans cells, cell surfaces of cortical thymocytes and interdigitating dendritic cells	+
CD-68	Monoclonal mouse antibody specificity for human CD-68 antigen, dako	1:10,000	Langerhans cells, mononuclear phagocyte system	+
CD45RO	Monoclonal mouse antibody specificity for human CD45RO antigen, dako	1:800	T lymphocytes, CD4 lymphocytes and thymocytes	+
Pan-cytokeratin	Monoclonal mouse antibody anti-human cytokeratin, novocastra	1:400	Human cytokeratin human, identification of cells of simple and stratified epithelial origin	−
Melanoma marker (HMB45)	Lyophilized monoclonal novocastra	1:200	Retinal epithelium, melanocytes and non-melanocytic cells	−
Melanina A	Lyophilized monoclonal; novocastra	1:200	Cytotoxic T lymphocytes	−

*+* Positivity

*−* Negative

## Discussion

Histiocytosis comprises a diverse group of proliferative disorders characterized by the infiltration and accumulation of histiocytes and other immune effector cells within various tissues [2]. Historically, the nomenclature regarding this entity has been confused. The disease had been subcategorized based upon different clinical manifestations, such as Histiocytosis X, Letterer-Siwe disease, Hand-Schuller-Christian syndrome and Eosinophilic Granuloma of bone. The Histiocyte Society proposed the reclassification of histiocytosis based upon the predominant cell type within the infiltrate. The classification system includes four divisions: dendritic disorders (including LCH), macrophage-related disorders, malignant histiocytic disorders and dendritic cell or macrophage-related histiocytic sarcoma [2, 3]. The LCH etiology has been discussed for many years and its reactive nature is suggested. Clonal proliferation has been reported with the presence of BRAF V600E oncogenic mutation in more than half of patients with LCH, which suggests a neoplasm [4, 6]. LCH is a rare disease with a wide variety of clinical presentations. Its course may damage different tissues, including the periodontal bone and gingiva. In addition, one of the types of LCH can lead to death [4, 5]. In our specific case, the patient had a lesion that mimicked periodontal disease associated with an oral squamous cell carcinoma or carcinomatous lesion.

LCH in adults has been reported at an incidence of 1:1,000,000 and a mean age of presentation of 34 years and is usually a multisystem disease. Childhood LCH has a reported incidence of 1:200,000, peaking between ages 1–4 years [3, 6]. In children, an association between high concentrations of salivary IL-1 β and PGE(2) and advanced stages of LCH have been observed, suggesting that the abnormal amount of these factors in the saliva may be a risk marker for disease progression [7].

Our case was distinct to most reported cases in the literature, since the patient was a 63-year-old man who did not show any bone or organ involvement other than the maxilla. Despite the strong possibility of pulmonary manifestation, due to the duration time of time that he had been a smoker, there was no evidence of pulmonary involvement. The chest x-ray confirmed this fact. However, experimental studies have shown that cigarette exposure is associated with pulmonary inflammation [8]. Interestingly, most LCH bone lesions that have been described in the literature were present in the skull, spine, or mandible [9]. In our case, the lesion was located only in the maxilla. Bone scintigraphy was determinant to show the unique bony involvement.

Diabetes insipidus (DI) is the most common endocrine abnormality, reported in 5–50 % of patients with LCH. DI is a result of the destruction of more than 80 % of the paraventricular-supraoptic neurons [10, 11]. Despite the high frequency of DI reported in prior literature, in our case, all hematological and biochemical parameters, liver function tests, urine osmolarity and endocrine evaluations were within the normal limits.

It is essential to note that the clinical and radiographic presentation of LCH could mimic an acute or chronic periodontal or periapical lesion, radicular cysts, osteomyelitis and malignancies. The lesions often appear as sharply punched-out radiolucent images, and the teeth appear to be “floating” when extensive alveolar involvement occurs [12]. Professionals should consider LCH as a possible diagnosis in different situations: periodontal lesions recurrent after conventional treatment, the presence of systemic symptoms previously mentioned and associated with periodontal lesions and after rapid, severe and localized periodontal bone loss. In this specific case, the lesion resembled a periodontal lesion associated with a carcinomatous lesion; however, the histological findings did not show any evidence of neoplastic cells.

The presence of pale histiocytes was fundamental to decide to do an immunohistochemical panel to investigate the nature of these cells. Monoclonal antibodies for S-100 (Fig. 3a), CD1a pan-cytokeratin, CD-68, melanoma marker (HMB45) and melanoma A protein were included in this panel. Positive staining for S-100, CD1a, and CD-68 established the final diagnosis in our case. The CD1a marker is essential for distinguishing Langerhans cells from other dentritic cells and histiocytes [13]. In our case, this additional examination by immunohistochemical techniques was critical for the establishment of the correct diagnosis of LCH and treatment planning for the patient.

The first step for the treatment of LCH should be to determine the number of organ systems involved. Then patients who have single-system disease should be subcategorized based on the number of sites involved. The presence or absence of organ dysfunction is a subcategory for patients who have multi-organ disease [14–18]. Curettage is generally sufficient for patients with localized bone lesions. However, it also possible to employ intralesional steroids or low-dose radiation. Treatment of multiorgan disease is controversial. Some state that high-dose prednisone should be the first-line therapy, whereas others suggest the use of single-agent chemotherapy [3, 14–17]. A combination of vinblastine and prednisone is an effective therapy used as the standard regimen for multisystem LCH in children and should be further explored in adult patients [18]. Other treatments, such as targeted therapy with BRAF inhibitors, has been used in select patients with encouraging results [18]. Another model indicates both BRAF mutation and IL-1 loop regulation as potential LCH therapeutic targets [19].

In our case, the patient underwent surgical extraction of all superior teeth, due to their periodontal involvement; curettage of the bone lesion; local radiation and corticoid therapies and prosthesis. The prognosis of LCH depends on the number of organs involved, as well as the presence of organ dysfunction and, in minors, on the age of the patient at the disease onset [4, 14–18]. In this specific case reported, the prognosis was favorable, due to single bone involvement, early diagnosis and proper treatment. Moreover, the patient had no signs of recurrence after 8 years of follow-up.

## Final considerations

In summary, we reported an unusual case of LCH in an elderly male patient who had a unique oral manifestation. LCH is a disease with a wide spectrum of clinical manifestations and a definitive diagnosis relies on biopsy and immunohistochemical examinations. It is extremely important that the doctor or dentist perform the correct diagnosis to plan the most appropriate treatment, since LCH clinically resembles other diseases that are treated differently. Moreover, the wrong diagnosis and treatment may compromise the patient’s life.

## Consent

The patient consented to the publication of this case report and any accompanying images.

## Abbreviation

LCH: langerhans cell histiocytosis
